# Endoplasmic Reticulum Stress and Tumor Microenvironment in Bladder Cancer: The Missing Link

**DOI:** 10.3389/fcell.2021.683940

**Published:** 2021-05-31

**Authors:** Zhenyu Nie, Mei Chen, Xiaohong Wen, Yuanhui Gao, Denggao Huang, Hui Cao, Yanling Peng, Na Guo, Jie Ni, Shufang Zhang

**Affiliations:** ^1^Central Laboratory, Affiliated Haikou Hospital of Xiangya Medical College, Central South University, Haikou, China; ^2^Cancer Care Center, St. George Hospital, Sydney, NSW, Australia; ^3^St George and Sutherland Clinical School, Faculty of Medicine, UNSW Sydney, Sydney, NSW, Australia

**Keywords:** endoplasmic reticulum stress, unfolded protein response, tumor microenvironment, bladder cancer, therapeutic target

## Abstract

Bladder cancer is a common malignant tumor of the urinary system. Despite recent advances in treatments such as local or systemic immunotherapy, chemotherapy, and radiotherapy, the high metastasis and recurrence rates, especially in muscle-invasive bladder cancer (MIBC), have led to the evaluation of more targeted and personalized approaches. A fundamental understanding of the tumorigenesis of bladder cancer along with the development of therapeutics to target processes and pathways implicated in bladder cancer has provided new avenues for the management of this disease. Accumulating evidence supports that the tumor microenvironment (TME) can be shaped by and reciprocally act on tumor cells, which reprograms and regulates tumor development, metastasis, and therapeutic responses. A hostile TME, caused by intrinsic tumor attributes (e.g., hypoxia, oxidative stress, and nutrient deprivation) or external stressors (e.g., chemotherapy and radiation), disrupts the normal synthesis and folding process of proteins in the endoplasmic reticulum (ER), culminating in a harmful situation called ER stress (ERS). ERS is a series of adaptive changes mediated by unfolded protein response (UPR), which is interwoven into a network that can ultimately mediate cell proliferation, apoptosis, and autophagy, thereby endowing tumor cells with more aggressive behaviors. Moreover, recent studies revealed that ERS could also impede the efficacy of anti-cancer treatment including immunotherapy by manipulating the TME. In this review, we discuss the relationship among bladder cancer, ERS, and TME; summarize the current research progress and challenges in overcoming therapeutic resistance; and explore the concept of targeting ERS to improve bladder cancer treatment outcomes.

## Introduction

Bladder cancer is the fourth most common and eighth most lethal malignant tumor in men in the United States, accounting for an estimated number of 81,400 new cases and 17,980 deaths in the United States in 2020 (Siegel et al., 2020). In China, a total of 80,500 new cases and 32,900 deaths were recorded in 2015 (Chen et al., 2016a).

Bladder cancer represents a broad spectrum of diseases, from low-risk, non-invasive lesions to advanced, muscle-invasive tumors. For non-muscle-invasive bladder cancer (NMIBC), although the survival rate is favorable, patients with low and intermediate risk have 5-year recurrence-free survival rates of only 43 and 33%, respectively (Ritch et al., 2020). For muscle-invasive bladder cancer (MIBC), although neoadjuvant chemotherapy provides a significant survival benefit, metastasis remains a devastating problem in a high portion of MIBC cases (50–70%), resulting in a dismal 5-year overall survival (OS) rate of 4.8% (Alfred-Witjes et al., 2017). Thus, novel treatment strategies against aggressive and advanced bladder cancer are clearly needed.

In eukaryotic cells, the synthesis and processing of secreted and membrane proteins take place in the endoplasmic reticulum (ER). The stability of its environment is a prerequisite for the successful synthesis and correct folding of proteins. When unfolded or misfolded proteins accumulate abnormally in the ER, they cause a harmful situation called ER stress (ERS) and usually trigger an intracellular signaling pathway called unfolded protein response (UPR) to restore normal ER protein-folding functionality. If this function is not restored, apoptosis is activated (Clarke et al., 2012). As such, several reports described a multi-faceted and paradoxical role of ERS in various diseases, such as neurological disorders, immune diseases, and cancers (Forouhan et al., 2018; Siwecka et al., 2019; Qin et al., 2020). In particular, whether ERS and UPR prevent or promote tumor growth has been hotly debated and warrants a careful review.

The tumor microenvironment (TME) refers to the environment in which tumor cells are located during tumorigenesis, development, and metastasis. Various cellular components (e.g., fibroblasts and immune cells) and non-cellular components (e.g., the extracellular matrix and physicochemical factors) can act on tumor cells and directly or indirectly regulate their tumorigenic, metastatic, and therapeutic resistance capacities. However, tumor cells can also alter or reshape the TME through autocrine or paracrine effects (Rodvold et al., 2017). Multiple stressors within the TME can cause ERS in tumor cells. They include intrinsic tumor attributes, such as hypoxia, oxidative stress, and nutrient deprivation and external stressors, such as chemotherapy, radiation, and immunotherapy. Cancer cells then utilize effective pathways to respond, adapt, and save themselves from ERS-induced cell death (Wouters and Koritzinsky, 2008; Saito et al., 2009; Tsachaki et al., 2018).

In this work, we review the ERS pathway and its role in bladder cancer; discuss the relationship of bladder cancer, ERS, and TME; highlight the significance of ERS in innate tumoricidal immune response and the efficacy of cancer immunotherapy; summarize the current research progress and challenges in this field; and explore the concept of targeting ERS to improve bladder cancer treatment outcomes in the clinical setting.

## ERS and Related Signaling Pathways

As mentioned above, multiple physiological and pathological stimuli can cause ERS, thereby triggering UPR. When a mild to moderate (yet persistent) ERS occurs, cells will cause transcriptional and translational changes through homeostatic UPR (hUPR), which promotes cell adaptation and enhances cell survival. As the ERS progresses to a degree where hUPR is inadequate to restore homeostasis, the UPR in the cell will be dominated by terminal UPR (tUPR). This process will actively initiate cell apoptosis to prevent continuous cell damage (Abern et al., 2013; Wallis et al., 2016).

**Figure 1:**
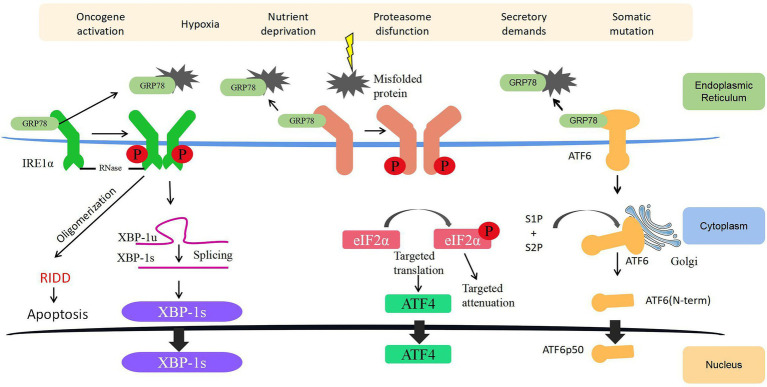
Endoplasmic reticulum (ER) stress (ERS) and unfolded protein response (UPR) signaling. Three ERS sensors (namely, IRE1α, PERK, and ATF6) collectively coordinate UPR signaling. Under normal conditions, GRP78 is attached to ERS sensors rendering them inactive. During ERS, GRP78 dissociates from the three transmembrane proteins on the ER membrane and activates these pathways. Together, the IRE1α, PERK, and ATF6 pathways regulate several genes with the ultimate goal of reinstating ER homeostasis and induce survival, angiogenesis, metastasis, and cell death resistance in cancer. ATF4, activating transcription factor 4; ATF6, activating transcription factor 6; ER, endoplasmic reticulum; eIF2α, eukaryotic initiation factor 2α; GRP78, glucose-regulated protein 78; IRE1α, inositol-requiring enzyme 1; P, phosphorylation; PERK, protein kinase-like ER kinase; RIDD, regulated IRE1α-dependent decay; S1P, Site1 Protease; S2P, Site2 Protease; XBP-1s, spliced X-box binding protein 1; and XBP-1u, unspliced X-box binding protein 1.

Unfolded protein response relies on signaling cascades mediated by three different transmembrane proteins localized on the ER membrane, namely, inositol-requiring enzyme 1α (IRE1α), protein kinase-like ER kinase (PERK), and activating transcription factor 6 (ATF6; Figure 1). When the ER is in a homeostatic state, a chaperone protein called glucose-regulated protein 78 (GRP78) in the ER binds to the intraluminal domains of the three transmembrane proteins and keeps them inactive. When a large number of misfolded and unfolded proteins accumulate in the ER, the three transmembrane proteins will be dissociated from GRP78 and activate three parallel UPR signaling pathways to reduce the burden caused by unfolded or misfolded proteins in the ER. They work by decreasing translation to reduce the folding requirements of newly synthesized proteins or guiding unfolded proteins into the cytoplasm for ubiquitination and destruction through the ER-associated degradation (ERAD) pathway (Ri, 2016; Li et al., 2019).

### IRE1α Pathway

IRE1 is a dual-effect protein with endoribonuclease (RNase) and serine/threonine kinase activities. Once the RNase domain of IRE1α is activated, it catalyzes a splicing reaction of X-box binding protein-1 (XBP1) mRNA and generates a resultant splicing variant called XBP1s. XBP1s upregulates the expression of many genes related to UPR, including *ZNF64*, *GPR7*, *PLK*, and *MRP*S22 (Ron and Walter, 2007; Peschek et al., 2015), to increase the expression of chaperones and foldase to mitigate ERS. In addition, under persistent and unresolved ERS, XBP1s can initiate a global mRNA degradation to limit the translation of proteins on the ER – a process known as regulated IRE1α-dependent decay, which ultimately induces ER destruction and apoptosis (Tam et al., 2014; Rashid et al., 2017). In bladder cancer, upregulated IRE1α is associated with an immediate UPR to restore protein homeostasis, and if UPR fails to alleviate ERS under prolonged or severe ERS, cells activate apoptosis pathways to eliminate damaged cells (Wu et al., 2019a). On the serine/threonine kinase front, phosphorylated IRE1α activates c-Jun N-terminal kinase (JNK) and NF-κB signaling, regulating a diverse range of cellular processes, such as inflammation, cell proliferation, survival, angiogenesis, and autophagy (Urano et al., 2000; Zhang and Kaufman, 2008; Han et al., 2009).

### PERK Pathway

The N-terminal of PERK inside the ER lumen is a stress-sensitive domain that binds to GRP78, whereas its C-terminal region on the cytoplasmic side contains a serine/threonine protein kinase domain that is capable of quickly and effectively inhibiting cell protein translation by phosphorylating the serine 51 on eukaryotic initiation factor 2α (eIF2α) upon activation by ERS. Phosphorylated eIF2α is essential for the translation of some UPR-dependent mRNAs through the upstream open reading frame, including activating transcription factor 4 (ATF4). Subsequently, ATF4 has several important target genes, such as the Growth Arrest and DNA Damage-Inducible Protein (GADD34) and C/EBP homologous protein (CHOP), which facilitate cell apoptosis (Lu et al., 2004). In bladder cancer, ERS activates CHOP and GADD34, which then trigger early apoptotic changes, including the dimerization of pro-apoptotic protein BAX (Zhang et al., 2011). On the other hand, the knockdown of CHOP in bladder cancer can partly reverse the pro-apoptotic effect exerted by cytotoxic drugs through blocking the translocation of BAX from the cytoplasm to the mitochondria (Zhang et al., 2018).

### ATF6 Pathway

Activating transcription factor 6 has two subtypes, namely, ATF6α and ATF6β. After ERS is activated, ATF6 on the outer side of the ER membrane will be packaged into the transporter and shuttled to the Golgi apparatus. In the Golgi apparatus, Site1 and Site2 Protease (S1P and S2P) will process ATF6 into an active ATF6p50 transcription factor and transport to the nucleus to bind and activate the promoter of UPR target genes (Yamamoto et al., 2004). In addition, ATF6 can achieve specific biological effects by regulating the expression of other transcription factors, such as activating the transcription of CHOP to induce cell apoptosis or activating the unedited expression of XBP1 linked to the IRE1α pathway (Walter et al., 2018). In bladder cancer, Zhang et al. (2021) found that deubiquitinase otubain 1 facilitates bladder cancer progression by inhibiting the ubiquitylation of ATF6 signaling, thereby remodeling the stressed cells through transcriptional regulation.

### Association Between UPR Pathway and Classical Signaling Pathways

Mounting evidence have suggested that the genetic alterations of classical cellular signaling pathways can also trigger ERS, and the three parallel yet distinctive UPR pathways interplay with them to determine oncogenic transformation and cell fate. For example, in brown adipocytes of hepatocellular carcinoma (HCC), the inhibition of the PI3K/AKT pathway leads to decreased levels of PERK phosphorylation; downregulates the expression of ATF4 and CHOP; decreases the phosphorylation levels of IRE1, GRP78, and XBP1; and antagonizes the effects of the ERS inducer tunicamycin (Winnay et al., 2020). In addition, PI3K/AKT has been found to positively regulate UPR in a lung fibrosis model (Hsu et al., 2017). During hypoxia, PERK can also be activated as a direct target of AKT (Blaustein et al., 2013). In addition, the activation of PERK can induce cellular autophagy by inhibiting the AKT/TSC/mTOR pathway (Blaustein et al., 2013). However, in the U87 glioblastoma cell line and SKBR3 breast cancer cell line, the activation of PI3K/AKT leads to the inactivation of PERK and its downstream eIF2α, thereby inhibiting the protective effect of PERK/eIF2α on tumor cells (Mounir et al., 2011). This indicates that the role of UPR is highly dynamic and depends on cells and conditions. In addition, the UPR pathway interacts with the MEK/ERK pathway during ERS. For example, in HCC cell lines (HEP3B and SMMC-7721), ERS inhibits AKT activity, allowing the activation of the MEK/ERK pathway and causing cell proliferation (Dai et al., 2009). IRE1 also activates ERK1/2 (Darling and Cook, 2014), and the inhibition of the MEK/ERK pathway in the breast cancer cell line U0126 renders cancer cells to become more sensitive to ERS-induced apoptosis (Yang et al., 2016). In MAPK-related pathways, UPR can regulate all three signaling axes, including JNK, p38, and ERK1/2. For example, IRE dimers can bind to ASK1 and phosphorylate to activate MKK4/7, thereby activating JNK (Urano et al., 2000). The JNK pathway is usually thought to be associated with apoptosis, but JNK can mediate c-Jun phosphorylation, thereby promoting cell survival (Darling and Cook, 2014). In addition, the activation of the IRE/JNK pathway induced by ERS can bind to Beclin-1 and regulate the occurrence of protective autophagy in cells (Kania et al., 2015; Senft and Ronai, 2015; Lin et al., 2019). ASK1 can also activate MKK3/6, which activates p38, ultimately causing an increase in p38-induced ATF6 expression and activation, as well as CHOP activation, leading to apoptosis (Luo and Lee, 2002).

In summary, obtaining an in-depth understanding of the complex network of oncogenic signaling and the key UPR factors is important, and the existence of this potential complementary and compensatory mechanism needs to be taken into consideration when targeting URP.

## Roles of ERS in Bladder Cancer

### ERS and Bladder Cancer Cell Proliferation

Instead of responding to the growth control system, cancer cells grow and divide in an uncontrolled manner, leading to continual unregulated cell proliferation and tumor growth. Rapidly proliferating cells require rapid protein synthesis and ER replication for division. As reviewed earlier, the fate of cells undergoing ERS depends on the intensity and duration of the stress, which will result in a pro-survival or pro-apoptotic effect on cancer cells.

Many studies have proved that ERS closely regulates the proliferation of bladder cancer cells. The ER-related degradation protein-1 (Derlin-1) is a core protein of the ER degradation pathway that can interact with a variety of proteins. Derlin-1 can form a protein complex with protein containing valine (p97), ubiquitin ligase, and ubiquitin protein. Then, it reverses the ERS by co-regulating substrate protein with major histocompatibility complex I and mediates the degradation of unfolded or misfolded proteins (Iida et al., 2011; Christianson and Ye, 2014; Mehnert et al., 2014). Therefore, the increased expression of Derlin-1 can make tumor cells more resistant to ERS. Wu et al. (2016b) showed that the expression of Derlin-1 in bladder cancer tissue is significantly higher than that in adjacent tissues, and its expression is positively correlated with tumor stage, histological grade, lymph node involvement, and muscle invasiveness. The mechanism behind this process (Wu et al., 2016b) may be due to the fact that the overexpression of Derlin-1 can upregulate the expression of matrix metalloproteinase (MMP)-2/MMP-9, and it can also cause extracellular regulated protein kinase (ERK) phosphorylation (Dong et al., 2017a). In addition, Wu et al. (2019a) found that extracellular vesicles (EVs) from bladder cancer cells can induce malignant transformation of susceptible cells adjacent to cancer by stimulating UPR during ERS and inflammation and promoting the proliferation, progression, and recurrence of bladder cancer. This study proposed novel mechanisms of EV-mediated tumorigenesis and ERS initiation (by horizontal transfer of the EV cargo), providing a novel insight into the mechanisms underlying bladder cancer carcinogenesis and recurrence.

### ERS and Bladder Cancer Cell Apoptosis

As discussed above, UPR alleviates ERS by suppressing protein synthesis and reinforcing the degradation of unfolded proteins. However, if the stress is beyond the capacity of the adaptive machinery, the cells will undergo apoptosis *via* several tUPR-mediated mechanisms. IRE1α activates JNK and p38-MAPK pathways that promote apoptosis (Ron and Hubbard, 2008). Moreover, p38-MAPK can activate the transcription factor CHOP, which enhances the expression of pro-apoptotic genes such as Bim while reducing the expression of Bcl-2 (Puthalakath et al., 2007). PERK attenuates mRNA translation under ERS by phosphorylating eIF2α, thereby inhibiting polypeptide chain synthesis. In addition, the phosphorylation of eIF2α activates ATF4, followed by CHOP and GADD34 (Lu et al., 2004).

Many drugs exert their tumor-killing effects by regulating ERS-related apoptosis pathways in bladder cancer. Thymoquinone, the major active compound of black seed oil, exhibits cytotoxicity to bladder cancer cells and induces apoptosis by upregulating the phosphorylated eIF2α, IRE1, and CHOP (Zhang et al., 2018). Similarly, flaccidoxide-13-acetate, isolated from cultured soft coral *Sinularia gibberosa*, was found to provoke ERS and activate the PERK–eIF2α–ATF6–CHOP pathway, causing inhibitory effects against the invasion and migration of bladder cancer cells (Wu et al., 2019b). Yuan et al. (2013) found that exposure to licochalcone A (a licorice chalconoid) could induce apoptosis in T24 bladder cancer cells by enhancing GRP78 and CHOP expression.

Euchromatic histone-lysine N-methyltransferase 2 (EHMT2) is an important enzyme in the process of histone modification. It is highly expressed in a variety of malignant tumor tissues, including bladder cancer and promotes tumor cell proliferation and invasion (Milner and Campbell, 1993; Tachibana et al., 2001; Huang et al., 2010; Mund and Lyko, 2010; Cho et al., 2011). BIX-01294 is a specific inhibitor of EHMT2 and has been found to have an inhibitory effect on bladder cancer (Kim et al., 2013). Cui et al. (2015) found that BIX-01294 stimulates ERS and triggers UPR by upregulating the expression of DDIT3, which ultimately causes bladder cancer cell apoptosis. Interleukin-24 (IL-24) is a unique IL-10 family cytokine that can selectively induce cancer cell apoptosis without damaging normal cells (Gupta et al., 2006). Recently, some researchers have proved that IL-24 exerts its cytotoxic effect through ERS-induced tumor cell apoptosis (Zhang et al., 2016b).

Collectively, these pieces of evidence indicated a convergent role of ERS in regulating cancer cell apoptosis, and these ERS-related pathways can be manipulated to exert therapeutic effects.

### ERS and Autophagy in Bladder Cancer

Autophagy is a highly conserved lysosomal degradation pathway that plays an essential role in the maintenance of cellular homeostasis. The by-products and damaged organelles produced by the metabolism of various biochemicals in the cell are swallowed by autophagosomes and transported to lysosomes, where they are degraded and recycled (Klionsky et al., 2021). Similar to other cancers, the role of autophagy in bladder cancer is double-sided (Santoni et al., 2013; Kou et al., 2017; Hua et al., 2018; Li et al., 2018; Schlütermann et al., 2018; Wang et al., 2018). On the one hand, Yang et al. (2018) found that BNIP3, a pro-apoptotic protein that belongs to the Bcl-2 family, can be activated by hypoxia-inducible factor-1α (HIF-1α) under hypoxic conditions and lead to autophagy initiation, which counteracts gemcitabine-induced apoptosis. On the other hand, Li et al. (2018) revealed that NVP-BEZ235, a dual PI3K/mTOR inhibitor, leads to cell death in cisplatin-resistant bladder cancer through autophagic flux activation without inducing apoptosis.

As autophagy is a stress-induced cellular mechanism, it would not be surprising to discover crosstalk between autophagy and ERS. The intertwined molecular mechanisms may vary: Ogata et al. (2006) indicated that ERS triggers autophagy *via* the IRE1–JNK pathway, but not the PERK or ATF6 pathway. However, other studies found that PERK/eIF2α phosphorylation triggers autophagy to adapt to ERS (Harding et al., 2000; Kouroku et al., 2007). Given the data above, several complex signaling pathways may contribute to the crosstalk between autophagy and ERS. Research has shown that ERS-mediated autophagy stimulates the occurrence and development of bladder cancer cells. For example, Liu et al. (2017a) observed a concurrent increase in the expression of ERS-related genes (ATF6, IRE1, EDEM1, and ERdj4) and autophagy-related genes (BECN, ATG3, and ATG5) in bladder cancer cells treated with melatonin and valproic acid. Although the anti-cancer activity of melatonin has long been considered to mediate ERS, melatonin is also an epithelial-mesenchymal transition (EMT) inhibitor, and some researchers have found that the attenuation of EMT signal in tumor tissues is closely related to melatonin-mediated ERS (Wu et al., 2016a; Yu et al., 2016b). Photodynamic therapy uses visible light and a light-absorbing agent to generate cytotoxic reactive oxygen species (ROS) within the tumor, which leads to tumor ablation. Buytaert et al. (2008) have recently used hypericin as a photosensitizer to effectively eradicate bladder cancer cells and subsequently found that autophagy-related genes WIPI1, MAP1LC3B, and ATG12 were upregulated, underpinning the involvement of autophagy in UPR in response to ERS in bladder cancer. Prolyl-4-hydroxylase subunit beta (P4HB) is an autophagy-related protein that is highly expressed in a variety of cancers including bladder cancer (Xu et al., 2014; Lyu et al., 2020). Co-expression network analysis and gene set enrichment analysis from two studies (Lyu et al., 2020; Wang et al., 2020) revealed that P4HB is involved in bladder cancer ERS response and associated with an unfavorable prognosis.

A pressing issue at the time of predicting whether the induction of ERS will activate autophagy in a protective or cytotoxic way is our relative lack of understanding of the molecular mechanisms through which autophagy regulates cell death. Therefore, the cellular context should be considered to understand how different ERS signals are integrated to yield a protective or cytotoxic autophagic response.

### ERS and Bladder Cancer Cell Resistance

Chemotherapy remains the mainstay of treatment for patients with muscle-invasive or metastatic bladder cancer (Flaig et al., 2020). Although cisplatin-based combinational chemotherapy is effective in tumor debulking, certain patients show initial response but progressively become unresponsive to the treatment. Therefore, exploring novel drug-resistance mechanisms to overcome chemoresistance is urgently needed for bladder cancer. Recently, an increasing number of chemotherapy resistance mechanisms involved in ERS have been discovered. In addition to its biological effects in promoting the proliferation and invasion of bladder cancer, the high expression of Derlin-1 can also induce bladder cancer cells to become resistant to cisplatin *via* the PI3K/AKT and MMP/ERK pathways. Lowering the expression of Derlin-1 could re-sensitize bladder cancer to cisplatin (Dong et al., 2017a). Gemcitabine is a cytosine analogue that exerts anti-tumor effects by interfering with the metabolism and synthesis of tumor cell genetic materials, which has been used as the first-line chemotherapy for bladder cancer (Schlack et al., 2016). Wang et al. (2020) found that the inhibition of P4HB, an ER chaperone, could sensitize bladder cancer cells to gemcitabine by activating apoptosis and the PERK/eIF2α/ATF4/CHOP pathways. Topoisomerase inhibitors, such as etoposide, can trigger programmed cell death through the caspase-dependent signal cascade in cancer cells (Schuler et al., 2000; Wang et al., 2020). Hence, they have been widely used to treat bladder cancer with a small-cell component histology in a neoadjuvant setting. GRP78 is a protein that binds to unfolded protein and triggers its degradation when ERS occurs. Its high expression can improve the tolerance of the ER to various stressors. Some researchers have found that high expression of GRP78 can cause bladder cancer cells to develop resistance to various topoisomerase inhibitors and protect them from apoptosis (Reddy et al., 2003).

## Crosstalk Between ERS and TME

**Figure 2:**
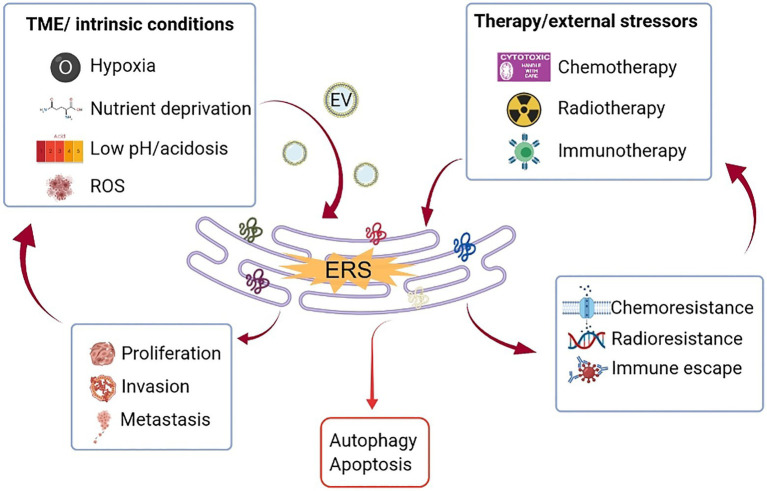
Crosstalk between ERS and TME. Uncontrolled tumor growth generates a hostile TME, characterized by hypoxia, nutritional deficiencies, and localized acidosis, which increase the accumulation of unfolded/misfolded proteins on the ER (partly mediated by EV) and consequently ERS. Therapeutic modalities, such as chemotherapy, radiation, and immunotherapy also trigger ERS. Depending on the intensity and magnitude of ERS, the cells face two different fates through the activation of UPR. If the stressor is persistent and strong that goes beyond the adaptive capacity, UPR will mediate cell death through the induction of apoptosis or autophagy. If the ERS is tolerated, UPR signaling will lead to tumor proliferation, metastasis, and treatment resistance, which in turn aggravate the hostility of the TME. ER, endoplasmic reticulum; ERS, endoplasmic reticulum stress; EV, extracellular vesicle; ROS, reactive oxygen species; TME, tumor microenvironment; and UPR, unfolded protein response.

Recent years have witnessed a shift in cancer research and therapeutic strategy from a cancer-centric model to a TME-focused one, as a substantial number of studies have proved that cellular and non-cellular components in the TME can reprogram tumorigenesis, invasion, metastasis, and response to anti-cancer therapies. On the other hand, the rapidly dividing cancer cells aggressively consume oxygen and glucose and discharge lactic acid waste, which affects the conditions of the TME. Cancer cells respond to stressors, such as hypoxia, nutrient deficiency, and ROS accumulation through a wide variety of mechanisms, one of which is the activation of UPR in ERS. In addition, as the major cellular constituents of the TME, immune cells have also been found to be altered and shaped by ERS, thus influencing the malignant transformation and progression of cancer cells (Figure 2).

### Hypoxia

Hypoxia is a common feature of the TME (Mazumdar et al., 2009; Jiang et al., 2015), and it can easily cause the accumulation of misfolded proteins as protein folding is an oxygen-dependent process, which makes ER sensitive to hypoxia (Koritzinsky et al., 2013). As UPR in ERS at the initial stages increases cancer cell survival and consequently the tumor mass, this may constitute a positive hypoxia–ERS–tumor growth–hypoxia feedback loop, further aggravating tumor proliferation. Hypoxia stabilizes HIF-1α and results in the activation of the PERK pathway of UPR through the phosphorylation of eIF2α and induction of ATF4 (Koumenis et al., 2002; Fels and Koumenis, 2006). In bladder cancer, HIF-1α has also been demonstrated to play a major role in mediating the cellular responses under low-oxygen conditions, such as promoting glycolysis (Zhang et al., 2016a; Xia et al., 2019), EMT (Lv et al., 2019), and autophagy (Yang et al., 2018), which contribute to tumor growth, invasion, and chemoresistance. Furthermore, hypoxia gene signatures have a strong and independent prognostic value for MIBC patients and can aid in the selection of patients for carbogen and nicotinamide treatment to reverse hypoxia to sensitize bladder cancer to radiotherapy (BCON Trial; Yang et al., 2017b). Of note, EVs that contain genetic materials have recently been recognized as an integral part in mediating the interaction and communication between cancer cells and the TME (Li and Nabet, 2019). On the one hand, the induction of ERS increases the biogenesis and release of EV through the IRE1α and PERK UPR pathways (Kanemoto et al., 2016); on the other hand, bladder cancer-derived EVs were found to activate UPR in ERS to promote malignant transformation (Wu et al., 2019a). Moreover, under hypoxia, bladder cancer cells secrete oncogenic long non-coding RNA-enriched EVs to remodel the TME, facilitating tumor growth and development (Xue et al., 2017). These observations offer novel perspectives as to the interaction and communication between hypoxia and ERS and open a new avenue for the development of targeted therapies, such as engineered EV therapeutics or EV-based small drug delivery.

### Nutrient Deficiency

The stability of the ER can be destabilized by nutrient deprivation. For example, uridine diphosphate-N-acetylglucosamine (UDP-GlcNAc) is synthesized by glucose and glutamine *via* the hexosamine biosynthetic pathway (HBP) and necessary for the correct folding of proteins in the ER and for the process of glycosylation. However, glucose or glutamine deficiency blocks the HBP, decreasing the synthesis of UDP-GlcNAc and increasing the precursor product N-acetylglucosamine (NAG). NAG can also bind to proteins in the ER, but triggers UPR because it does not drive the correct folding of proteins (Braakman and Bulleid, 2011; Denzel and Antebi, 2015; Domblides et al., 2018). In bladder cancer, the increase in NAG is induced by a limited supply of glucose, causing ERS and eventually a G2/M phase block (Isono et al., 2013). Similarly, amylo-α-1,6-glucosidase, 4-α-glucanotransferase (AGL), a key enzyme of glycogenolysis, was found to have an inhibitory effect on bladder cancer. Bladder cancer cells with deficient AGL expression have a higher glycolytic activity and glycine synthesis capacity than those with normal expression of AGL, and this capacity can greatly contribute to the proliferation of bladder cancer (Sun et al., 2019). Impaired amino acid metabolism can affect ERS through the action of the TME. Amino acid starvation can induce an integrative stress response (ISR), which is essential for tumor cells to adapt to stressors (Ritterson-Lew et al., 2015), making them resistant to chemotherapeutic agents. The induction of ISR in bladder cancer can cause resistance to the proteasome inhibitor, bortezomib (Pakos-Zebrucka et al., 2016). Interestingly, a high-fat diet can lead to alterations in the size, composition, and fluidity of ER membranes, which can affect functions such as protein glycosylation, thereby promoting ERS (Qi et al., 2013). The level of lipid-derived acetyl coenzyme A was found to be proportional to the proliferative capacity of bladder cancer cells, and excessive intake of acetyl coenzyme A synthase 3 and fatty acids is responsible for this phenomenon (Wei et al., 2006). Even in the presence of sufficient glucose, tumor cells prefer to use the glycolytic pathway to produce lactate for energy. At the same time, tumor cells competitively inhibit the sugar metabolism of immune cells in the microenvironment, thus creating a local acidic microenvironment. This microenvironment not only hampers the clearance of bladder cancer by immune cells, but also promotes tumor proliferation and invasion by fostering neovascularization (Afonso et al., 2020; Zhang et al., 2020).

### Reactive Oxygen Species

The accumulation of intracellular ROS can significantly affect the state of proteins within the ER lumen (Dong et al., 2017b). For example, limiting the amount of intracellular glutamine can induce ERS by disrupting glutathione production and thus altering the redox state within the ER lumen (Shimizu and Hendershot, 2009). In bladder cancer, glutamine can promote its proliferation by increasing ROS and regulating the expression of signal transducer and activator of transcription 3 (STAT3; Zhang et al., 2017a). The inner mitochondrial membrane is the main site of ROS production. For example, during β-oxidation of fatty acids, large amounts of ROS are subsequently produced as by-products of the electron transport chain. In addition, some cytokines or growth factors with pro-inflammatory effects can lead to the continuous activation of NADPH oxidases (NOXs), producing large amounts of ROS (Choudhary et al., 2011). The constant activation of NOX, the accumulation of intracellular ROS, and the depletion of glutathione can jointly affect the caspase activity of bladder cancer cells and regulate their apoptosis (Liou and Storz, 2010).

### Tumor-Associated Immune Cells

Bacille Calmette–Guérin (BCG) therapy is the current first-line treatment for high-risk NMIBC patients. With this in mind, bladder cancer was one of the earliest cancers where immunotherapy was first utilized. The protective effect of BCG is mediated by increasing the number of macrophages in the TME, urinary bladder wall surrounding the tumor, and urine (Fuge et al., 2015). Tumor-killing macrophages (M1 type) are the main enforcers of the anti-tumor effect of BCG, whereas M2 macrophages may negatively influence the immune response of bladder cancer to BCG (Liu et al., 2019). However, most of the tumor-associated macrophages (TAMs) in bladder cancer are polarized into M2 macrophages, which resist the anti-cancer effect of BCG (Shan et al., 2018; Kobatake et al., 2020), due to their suppressive immune response to cancer and pro-tumor progression (Aljabery et al., 2018; Asano et al., 2018). New and effective strategies are needed to reverse M2 polarization and redirect TAMs to become tumoricidal.

Currently, numerous studies have shown that ERS of tumor cells can influence tumor progression by altering the function of infiltrating immune cells in the TME. Bladder cancer cells subjected to ERS could infiltrate the tumor tissue by the excessive release of specific cytokines and recruitment of myeloid-derived suppressor cells (MDSCs; Zhang et al., 2017b). MDSCs can increase the resistance of bladder cancer cells to cisplatin and the checkpoint inhibitor α-PD-L1 antibody (Takeyama et al., 2020). In addition, MDSCs can cause inflammation and promote angiogenesis within the bladder cancer tissue (Dominguez-Gutierrez et al., 2020). On the other hand, IL-6 enriched in the TME can block MDSCs by triggering IRE1α–XBP1 signaling in macrophages through the activation of STAT3 and STAT6 (Yan et al., 2016; Yang et al., 2017a). ERS-related markers, such as GRP78, ATF6, PERK, and IRE1α can recruit CD68+ macrophages to infiltrate the peritumor tissue and upregulate PD-L1 expression in macrophages *via* EVs, which subsequently inhibit T-cell function and facilitate immune escape (Liu et al., 2019; Xue et al., 2019).

ERS is commonly believed to affect natural killer (NK) cell-dependent tumor recognition (Obiedat et al., 2019), and NK cells are mainly regulated and recruited by IRE1α–XBP1 signaling (Dong et al., 2019) and mediate bladder cancer cell differentiation or death (Ramakrishnan et al., 2019). However, bladder cancer-infiltrating NK cells have a functional defect: they are unable to complete the degranulation process, resulting in the inability to exercise their cytolytic effect; by contrast, circulating NK cells do not have this functional defect (Tsujihashi et al., 1989). BCG may repair this defect and restore the function of NK cell degranulation (Kleinnijenhuis et al., 2014; García-Cuesta et al., 2015).

T cells are indispensable immune cells in the immune system, and ERS occurring in bladder cancer has been shown to modulate T-cell-mediated cancer cell proliferation, metastasis, and sensitivity to immunotherapy (Chugh et al., 2013; Pommier et al., 2018; Tao et al., 2018). Studies have found that T cells playing various roles are recruited into the bladder cancer TME during disease, including pro-inflammatory T cells (which may be beneficial to the body) and anti-inflammatory T cells (which may be detrimental; Oh et al., 2020; Wu and Abraham, 2021). Thus, regulating T-cell infiltration may be a promising therapeutic strategy to target bladder cancer.

Notably, various stressors in the TME can stimulate ERS not only in cancer cells but also in immune cells; for example, high levels of cholesterol in the TME can activate IRE1α–XBP1 signaling in T cells within cancer tissues, induce programmed death protein 1 (PD-1) expression, and prevent T cells from exerting tumor-killing effect (Ma and Yi, 2019). Furthermore, the accumulation of ROS in the TME promotes ERS in dendritic cells (DCs) and the sustained activation of IRE1α–XBP1, which subsequently inhibits their function of presenting local tumorigenic antigens to intratumoral T cells (Herber et al., 2010; Gao et al., 2015).

## Clinical Implications

### ERS as a Cancer Prognostic Marker

Endoplasmic reticulum stress-related molecular markers have been reported to have prognostic values for cancer patients. The roles of PERK and IRE1α signaling in cancer prognosis depend on cell types, stress conditions, and the TME and thus are inconsistent (Clarke et al., 2014). For example, the expression levels of ERS markers, such as GRP78, PERK, and IRE1α in human HCC tissues are proportional to CD63/PD-L1^+/+^ macrophage infiltration and predict poor clinical prognosis (Liu et al., 2019). Analysis of glioma patient datasets showed that the overexpression of PERK pathway signature is strongly correlated with chemotherapy resistance and poor OS (Del Vecchio et al., 2014). Similarly, another study revealed that high expression of ERS markers within DCs in human ovarian cancer tissues is associated with reduced T-cell infiltration (Cubillos-Ruiz et al., 2015). Song et al. (2018) also found that high expression of XBP1 (from IRE1α signaling) in T cells is associated with less T-cell infiltration and often observed in ascites, which only accumulates in patients with advanced or metastatic diseases. Furthermore, they found that low XBP1 expression exhibits excellent anti-tumor immunity, with a reduced tumor progression and prolonged OS in ovarian cancer mouse models (Song et al., 2018). However, in one study, XBP-1 isoforms were found to be differently associated with the outcome of breast cancer endocrine therapy: high levels of XBP-1u favor tumor cell apoptosis, whereas high levels of XBP-1s favor tumor survival. In bladder cancer, the overexpression of XBP1 has been found to correlate with the poor OS in transitional cell carcinoma patients (Chen et al., 2016b). In addition, ATF6 was also primarily recognized as a protective modulator in cancer during ERS. A growing body of literature has demonstrated that ERS-related ATF6 contributes to poor survival in different types of cancers, including colon cancer (Liu et al., 2018), glioblastoma (Dadey et al., 2016), prostate cancer (Liu et al., 2017b), and osteosarcoma (Yarapureddy et al., 2019).

### ERS and UPR as Therapeutic Targets

As aberrant UPR and ERS are major contributors to cancer development, chemoresistance, and poor prognosis, there has been strong interest in clinically influencing this process as a strategy to restrain tumor growth and reverse drug resistance. Two approaches can be used to target the ERS pathways: one being the inhibition of UPR-mediated adaptive responses to interrupt ER homeostasis, the other being the induction of sustained and lethal ERS that leads to cell death. Drugs that modulate the ERS or UPR, either as a monotherapy or in combination with chemotherapy, targeted therapy, and immunotherapy, have shown promising preclinical treatment efficacy and warrant further investigations and trials (Hetz et al., 2019).

#### IRE1α/XBP-1 Pathway

IRE1 RNase inhibitors (including B-I09, STF083010, MKC3946, and MKC8866) have shown good therapeutic performance in multiple myeloma (MM), breast cancer, prostate cancer, melanoma, lymphoma, and chronic lymphocytic leukemia (Tang et al., 2014; Logue et al., 2018; Xie et al., 2018; Zhao et al., 2018; Sheng et al., 2019; Jin and Saatcioglu, 2020). For example, MKC3946 significantly enhanced cytotoxicity induced by bortezomib (a proteasome inhibitor) in an MM xenograft model (Mimura et al., 2012). As a single agent, MKC8866 shows a significant tumor-suppressing effect; when used with chemotherapy such as paclitaxel and docetaxel, the combination shows surprisingly superior tumor-killing and survival-improving capabilities compared with the chemotherapy agent alone (Logue et al., 2018; Zhao et al., 2018). Of note, these inhibitors are capable of blocking the downstream XBP1 splicing without affecting the upstream IRE1α or PERK or ATF6 pathway, making them superior candidates for clinical trials with good tolerability, which explains that long-term usage of MKC8866 is effective in breast and prostate cancers in preclinical models, without causing substantial toxicity to normal tissues (Zhao et al., 2018; Sheng et al., 2019).

Another group of IRE1α inhibitors, IRE1α kinase inhibitors, also shows significant efficacy in an *in vivo* model of MM xenografts. IRE1α kinase inhibitor compound 18, also known as KIRA8 or AMG-18, inhibits the growth of MM and sensitizes the myeloma to current first-line therapeutic agents, bortezomib, and lenalidomide (an immunomodulatory agent; Harnoss et al., 2019). However, the suppression of XBP1s in MM was shown to induce bortezomib resistance *via* diminishing ER front-loading and cytotoxic susceptibility to inhibition of ERAD (Leung-Hagesteijn et al., 2013). This suggests that IRE1α inhibitors may trigger other UPR pathways *via* a feedback loop and may not fully recapitulate the effects of IRE1α gene ablation. More surprisingly, Auf et al. (2010) observed in a glioma mouse model that blockade of IRE1 reduces angiogenesis and tumor growth rate but causes extensive invasiveness and angiogenesis. Further investigation of IRE1α signaling is required to unveil the complex relationship between angiogenesis and invasiveness, and the development of highly selective inhibitors may therefore represent a more appropriate approach.

Chen et al. (2016b) discovered a novel natural product analogue CYD 6–17 that has a potent inhibitory effect on multidrug-resistant bladder transitional cell carcinoma by decreasing the binding of XBP1 to the promoter region. The delivery of CYD 6–17 significantly inhibited tumor growth using a xenograft model without detectable side effects.

#### PERK/eIF2α/ATF4/CHOP Pathway

Direct targeting of CHOP and ATF4 is challenging for conventional inhibitors as CHOP and part of ATF4 molecules are located in the nucleus, rendering the upstream transducer PERK and eIF2α the only viable options.

As a rationale, the inhibition of PERK signaling reversed the multidrug resistance of de-differentiated breast cancer cells (Del Vecchio et al., 2014), and the inhibition of the eIF2α-dependent arm of UPR reversed the tumor radioresistance in a subset of hypoxic glioblastoma cells (Rouschop et al., 2013). Similarly, the knockdown of ATF4 in combination with radiation led to reduced proliferation and colony formation in glioblastoma (Dadey et al., 2018).

On a pharmaceutical front, GSK2606414 and GSK2656157 are two of the most well-studied PERK kinase inhibitors. Of note, GSK2606414 was found to enhance PD-1 blockade efficacy in a sarcoma model (Hurst et al., 2019), and GSK2656157 showed tumor-killing and chemo-sensitizing effects in preclinical models of MM, pancreatic, and colon cancers (Atkins et al., 2013; Shi et al., 2019). In spite of the therapeutic efficacy, the use of GSK2606414 and GSK2656157 comes with significant “on-target” side effects such as diabetes caused by pancreatic β-cell loss (Yu et al., 2015). Moreover, both agents exhibit an “off-target” effect by showing a high inhibitory affinity to receptor-interacting kinase 1, which regulates pro-survival NF-κB signaling and cell death (Rojas-Rivera et al., 2017). The on-target side effects, along with the off-target effects, have largely hindered the clinical translation of GSK2606414 and GSK2656157.

eIF2α is located downstream of PERK and is a convergent node of ISR. The ISR inhibitor (ISRIB) is a potent eIF2α inhibitor that suppresses eIF2α phosphorylation (activation). It has shown a significant tumor-suppressing effect in prostate cancer when used alone in a mouse model *in vivo* (Nguyen et al., 2018) with no overall toxicity. Though ISRIB is difficult to formulate and insoluble given its high potency, it is nevertheless, a promising candidate for pharmaceutical exploration. Some natural compounds have shown treatment advantages in various cancers including bladder cancer *via* this pathway. Arctigenin (Kim et al., 2010), 11-epi-sinulariolide acetate (Lin et al., 2016), and flaccidoxide-13-acetate (Sinclair, 1988) have been proved to kill colon, cervical, or bladder cancer cells through the activation of the PERK/eIF2α/ATF4/CHOP pathway.

#### ATF6 Pathway

The pharmacological inhibition of ATF6 has not been vastly explored, and it may be attributed to the fact that this pathway relies on ATF6 protein alone to perform its function. Some investigators have found that ceapins, UPR inhibitors, can selectively block ATF6 signaling by impeding the translocation of ATF6α to the Golgi (Gallagher et al., 2016; Gallagher and Walter, 2016). Given that ATF6p50 is translocated to the nucleus once it is successfully sheared, it is difficult to make drugs that can stop ATF6p50 from functioning. However, the successful shearing of ATF6p50 depends on S1P and S2P, so the regulation of S1P and S2P can indirectly affect the function of ATF6. If the expression of S1P is inhibited using an inhibitor (PF-429242), it also reduces the expression of ATF6 and GRP78, which in turn activate IRE1α and PERK signaling, leading to apoptosis (Lebeau et al., 2018). Another herbal extract, baicalin, induces apoptosis through the targeted activation of S2P, and the effect is mitigated by the knockdown of ATF6 (Yu et al., 2016a).

Despite considerable efforts to improve BCG, no existing immunotherapy outperforms BCG for the treatment of high-risk NMIBC to date. Currently, five anti-PD-1/PD-L1 immunotherapeutic drugs (namely, atezolizumab, durvalumab, avelumab, nivolumab, and pembrolizumab) have been approved for the treatment of advanced and metastatic bladder cancer and demonstrated satisfactory efficacy (Powles et al., 2020). New approaches that incorporate emerging immunotherapies might successfully synergize with BCG to improve patient outcomes. In terms of ERS in immunotherapy, disabling ERS sensors or orchestrating UPR pathways can enhance anti-tumor immune responses. For example, Cubillos-Ruiz et al. (2015) found that knockdown of XBP1 in DCs enables the re-activation of CD8+ T cells and prolongs survival in a metastatic ovarian cancer mouse model. This may represent a novel notion to control UPR not only in cancer cells but also in immune cells within the TME for improving the efficacy of cancer immunotherapies. Of course, further studies are warranted to test these new ideas.

Interestingly, recent studies showed that several antiviral drugs, such as lopinavir, ritonavir, and nelfinavir, can inhibit the proliferation of bladder cancer cell line *in vitro* by inducing ERS (Sato et al., 2018; Okubo et al., 2019). However, this inhibitory effect on tumors may have nothing to do with the type of tumor and not be selective as these three drugs are potent inhibitors of proteases, which adaptive UPR relies on in response to ERS.

Overall, drugs that modulate ERS and UPR-related regulators have a great anti-cancer potential, and ERS-related markers are also important in predicting patient prognosis. The use of pharmacological inhibitors of UPR signaling may help to improve the prognosis of cancer patients. However, drugs that exert superior therapeutic potency and minimal side effects in bladder cancer are lacking. An important prerequisite of an ideal anti-cancer drug is that it should be non-toxic to or does not trigger ERS in normal cells. However, some active protein secretory cells, such as pancreatic β-cells mentioned above, require ERS as a rapid control mechanism and thus will have to be considered carefully. It is also worthwhile to test the synergistic effects of two single inhibitors or investigate the possibility of a dual inhibitor that targets two different UPR pathways to exert enhanced therapeutic potency.

## Conclusion

In bladder cancer, ERS and UPR are widely involved in multiple cellular processes and cell fate determination, including cell proliferation, autophagy, apoptosis, and therapeutic resistance. A growing body of literature suggested that UPR plays a cytoprotective and pro-oncogenic role in cancer to enable cancer cells to cope with adverse microenvironmental stimuli, such as hypoxia, nutrient deficiency, and ROS. This may represent a mechanism underlying the invasive, resistant, and recurrent behaviors of bladder cancer.

This review has focused on the crosstalk between ERS and TME and demonstrated its roles in bladder cancer and clinical implications. We also shed some light on the roles of EV in ERS–TME interaction and explored the concept of targeting ERS to revive innate tumoricidal immune response and enhance the efficacy of emerging cancer immunotherapy.

Unfolded protein response signaling also interacts with tumor regulatory genes (Rather et al., 2020) and signaling pathways (Oakes, 2020). Recent studies have also revealed that ERS contributes to several hallmarks of cancer (Limia et al., 2019; Pluquet and Galmiche, 2019; Martelli et al., 2020), but the mechanisms are poorly understood. Further studies are needed to elucidate the ambivalent roles of ERS responses in cancer and clarify the complex network between UPR and other signaling pathways. An in-depth understanding of such key players will facilitate the development of novel selective inhibitors with high potency and low toxicity to improve the current bladder cancer treatment.

## Author Contributions

ZN, JN, and SZ conceived and designed the framework of the study. MC, XW, YG, DH, HC, YP, and NG collected and reviewed the data. ZN wrote the manuscript. JN and SZ reviewed and edited the manuscript. All authors contributed to the article and approved the submitted version.

### Conflict of Interest

The authors declare that the research was conducted in the absence of any commercial or financial relationships that could be construed as a potential conflict of interest.
